# In Vivo Transcriptional Profiling of Human Pathogenic Fungi during Infection: Reflecting the Real Life?

**DOI:** 10.1371/journal.ppat.1005471

**Published:** 2016-04-14

**Authors:** Stefanie Allert, Sascha Brunke, Bernhard Hube

**Affiliations:** 1 Department of Microbial Pathogenicity Mechanisms, Leibniz Institute for Natural Product Research and Infection Biology, Hans-Knoell-Institute, Jena, Germany; 2 Center for Sepsis Control and Care, University Hospital Jena, Germany; 3 Friedrich-Schiller-University Jena, Germany; McGill University, CANADA

Multiple types of microbial infections in humans are caused by viruses, bacteria, fungi, or parasites. The outcome of these infections is largely determined by the genomes of the pathogen and host and the appropriate expression of their genes. As both host and microbe have to dynamically respond to changing conditions during the course of an infection, analyzing RNA profiles of the pathogen and the infected host can yield indispensable insight into the disease process. Such data can provide us with crucial information about (1) the mechanisms of host–microbe interactions; (2) factors that are specific to certain infection processes or stages and are, hence, potentially suitable for the development of advanced diagnostics (biomarkers); (3) factors or attributes essential for microbial growth, survival, or virulence in the host, and which are potentially exploitable as drug targets; and (4) nonprotective and protective immune responses applicable for immune therapies. In this review, we discuss the methodologies, technical challenges, potential pitfalls, and key biological messages obtained from studies dealing with transcriptional profiling of human pathogenic fungi during interactions with the host (in vivo) or ex vivo within organs (e.g., perfused organs) or host-derived fluids (e.g., cerebrospinal fluid [CSF]). We will focus on major fungal pathogens, such as *Candida*, *Aspergillus*, *Blastomyces*, *Arthroderma*, and *Cryptococcus* species, which account for millions of infections every year [1].

## Infection Models, Patient Samples, and Technical Challenges

**Fig 1:**
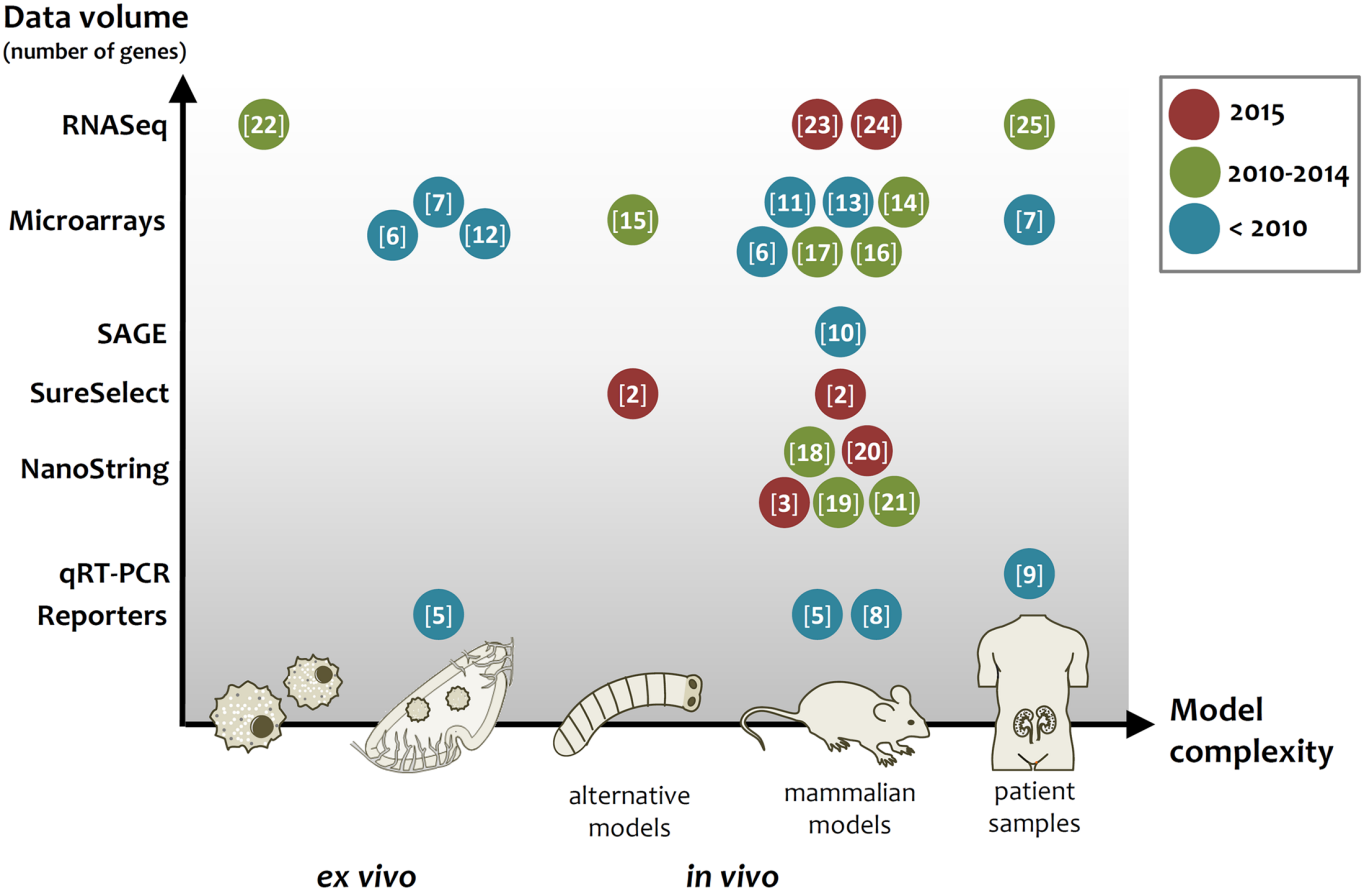
Infection models and gene expression data. This figure provides an overview of the models and technologies used in the studies reported in this review. Note that studies [5–7] used ex vivo and in vivo models, while study [2] compared an alternative with a mammalian model.

Different aspects of pathogenesis can be studied at the transcription level using different infection models. These can range from simple in vitro and ex vivo models via complex in vivo models to patient samples (Fig 1). Each model has specific advantages and disadvantages. For example, in vitro or ex vivo models can mimic distinct stages of infection, but lack the complexity of a living host. In general, the methodological difficulties increase with the complexity of the model, but so too does the potential outcome and relevance to the clinical setting.

The technical challenges for obtaining meaningful transcriptional profiling data from infection models are, however, manifold: foremost is the need for rapid RNA isolation, to avoid transcriptional changes and RNA degradation during the purification procedure, in addition to the low numbers of microbial cells within infected tissues (and, thus, high host-to-microbe RNA ratios) and the diverse cell populations during infection, to name only a few. Separating host and pathogen RNA after isolation can be similarly difficult, especially for eukaryotic pathogens that share the polyadenylation of mRNA with their hosts. However, RNA enrichment protocols have been successfully used [2,3].

Despite these technical challenges, an increasing number of excellent in vivo and ex vivo transcriptomics studies have allowed us to define both conserved and specific fungal infection strategies and have provided an invaluable first glimpse into conditions inside the host as experienced by the pathogen during infections [4].

## From Single-Gene Expression to Genome-Wide Dual RNA Sequencing

The simplest and probably most direct expression analyses from infection models or patients focus on few selected genes and are generally based on GFP reporters (“single cell profiling”) [5], In Vivo Expression Technology (IVET) [8], or quantitative reverse transcriptase PCR (qRT-PCR) (Fig 1) [9]. Important early findings were made using these techniques, including the heterogeneity of pathogen expression patterns within the same organ [5], the induction of the glyoxylate cycle and gluconeogenesis after phagocytosis of *Candida albicans* [5], or the infection-stage-specific and highly variable expression of individual members of a gene family in mice [8] and in patients [9]. All these studies allowed important first insights into the regulation of individual genes in actual infection situations.

Parallel expression analyses are technically more challenging, but can yield information on large numbers of genes (up to genome-wide). Such approaches include: Serial Analysis of Gene Expression (SAGE) [10], microarrays [6,7,11–17], NanoString [3,18–21], SureSelect [2], and RNAseq (Fig 1) [22–25]. All of these techniques require sufficient amounts of high-quality RNA and, generally, annotated genome sequences. The advantage of NanoString [3,18–21] is the very high sensitivity: no enrichment of pathogen cells is required. Similarly, SureSelect allowed transcript detection of >85% of all genes of *C*. *albicans* [2]. With the costs of sequencing becoming rapidly cheaper and bioinformatic pipelines constantly improving, RNASeq and related methods will likely be the technology of choice in the future, especially as it allows, in combination with sophisticated systems biology tools, dual- or even multi-species analyses: simultaneous, comprehensive transcriptional profiling of pathogen(s), host, and their interactions [22,23].

## Dissecting the Transcriptional Profiles of Complex In Vivo Models

Complex models can simulate the actual clinical infection, but generally produce highly complex transcriptional profiling data. These models, therefore, present a challenge not only for data analysis but also for their interpretation. Tools exist to reduce such complexities, e.g., gene ontology (GO) term or gene set enrichment analyses; however, one must be wary not to reduce the data to a descriptive inventory of known processes, with little new information for the infection process. If, however, the analysis and interpretation of transcriptional profiling data includes both hypothesis-driven and hypothesis-creating elements, in combination with sophisticated bioinformatics tools, important insights into biological processes and networks during an infection can be gained, as shown below.

One possible pitfall in interpreting in vivo transcriptional profiling is the fact that data are often average mRNA levels of mixed populations of cells co-isolated from very different environments (microniches) within the host (a problem that can be circumvented by the use of single-cell profiling; see above). This is presumably more important in vivo, as in laboratory cultures, and in simpler in vitro infection models, in which infecting fungi can at least be semi-synchronized. The dynamic processes of true in vivo infections, on the other hand, likely result in an almost individual transcriptional adaptation of each fungal cell or small cluster, which is more likely to be lost by this averaging. Therefore, the study design for in vivo transcriptional profiling should consider the spatiotemporal patterns of infections and should ideally include several different time points and carefully selected infection sites for analysis. So far, in vivo time series experiments are the exception rather than the rule [6,17], but with decreasing sequencing costs this will likely change in the near future.

The dissection of complex infection processes on the transcriptional level may also benefit from the inclusion of reductionism: transcriptional markers and patterns defined from in vitro experiments, indicative of particular physiological conditions, can be detected in complex in vivo datasets. For example, the *Aspergillus fumigatus* transcriptome during murine infections exhibited signatures also observed during in vitro iron and nitrogen starvation and adaptation to alkaline pH [11]. Similarly, SAGE analysis of *Cryptococcus neoformans* cells recovered from mammalian lungs were compared with data from cells grown in defined culture conditions, indicating that genes involved in alternative carbon metabolism are expressed in vivo [10]. Comparison of in vitro and in vivo transcriptomes may therefore allow conclusions about the microniches to which at least subpopulations of fungi are exposed during infection, allowing us to see the host through the eyes of the pathogen. However, while often very helpful, direct application of in vitro to in vivo data can, in some cases, also be misleading. For example, Pongpom et al. showed that the genes regulated by two *A*. *fumigatus* transcription factors differ strongly between in vitro growth and infections, with some virulence-associated genes being responsive only in vivo [20]. This is an important finding, as it shows again that established regulons of microbial transcription factors can be modulated by the conditions in the host. The same is true for *C*. *albicans*, in which, for example, Bcr1-responsive genes differ significantly in in vitro and in vivo biofilms, with very little overlap [19,26]. Dissecting complex in vivo data with simpler in vitro models, while useful, is therefore not always straightforward. However, the most interesting data may lie within the deviations from the expected results.

Another potential pitfall of data interpretation lies in the choice of the control dataset. For example, subtracting in vitro transcriptional data from in vivo data to identify infection-associated genes may remove crucial information. Although the identification of genes predominantly expressed in the in vivo setting is the foremost aim of most in vivo studies, genes expressed under laboratory conditions may also be critical for the infection process. For example, the yeast-phase-specific, important virulence gene *BAD1* of *Blastomyces* was not found as a specifically in vivo expressed gene [24]. Similarly, it was observed that the *C*. *neoformans* in vivo gene expression profiles in the CSF of infected patients were similar to those in complex culture growth medium rather than ex vivo CSF [25].

In summary, the road to a successful in vivo transcriptional profile leads via (1) an appropriate infection model (or patient sample), (2) a defined scientific question and hypothesis, (3) early consultation of bioinformaticians and (4) solid experimental design that considers the stage- and tissue-specific dynamics of infections, and (5) avoidance of potential pitfalls (e.g., in fungal cell enrichment procedures), which should lead to (6) high-quality pathogen and/or host RNA in sufficient amounts, allowing the use of (7) sophisticated systems biology tools for (8) hypothesis-driven and hypothesis-creating interpretation of transcriptional profiling data.

## Gene Expression during Infection: What Can We Learn from In Vivo Transcriptional Profiling?

Several studies have tackled these technical challenges and gained transcriptome data directly from sites of infection. So, what have we learned from these studies?

**Fig 2:**
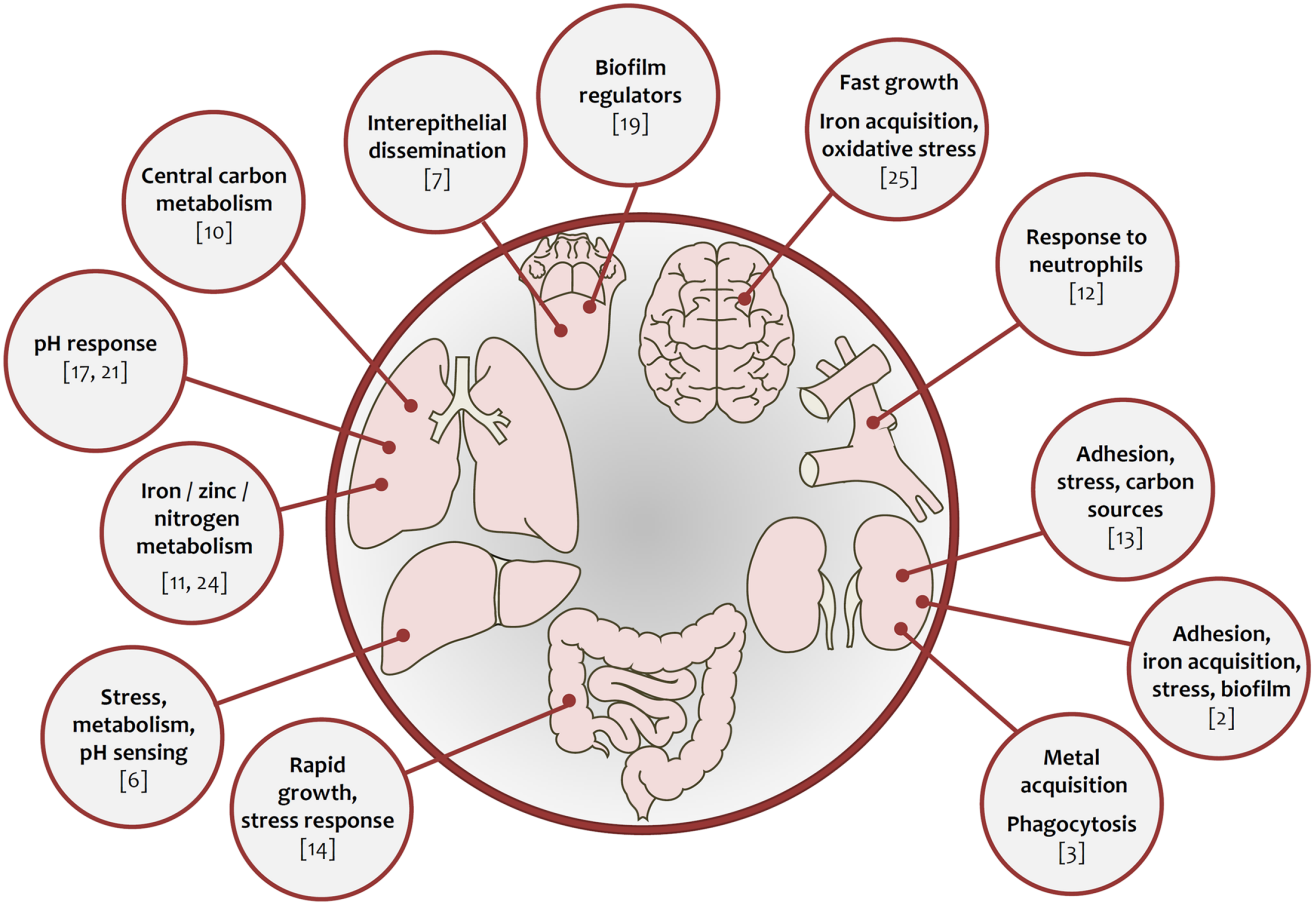
Fungal gene expression during infection. Key observations from studies discussed in this review.

In vivo single-cell profiling, IVET, qRT-PCR, or NanoString of selected genes have provided important and sometimes surprising insights (Fig 2): (1) the expression of metabolic pathways of fungi during systemic infection is niche-specific, (2) the expression of known virulence factors can depend on the type of infection, and (3) some of these factors are expressed in both infected mice and human patients [5,8,9,18].

Specifically, in an early use of microarrays and ex vivo blood infections, Fradin et al. confirmed the dominant role of neutrophils in clearing *C*. *albicans* blood infections [12]. Thewes et al. identified the central role of pH adaptation for liver invasion in mice and ex vivo perfused organs from pigs [6], Zakikhany et al. identified a regulator of interepithelial dissemination by using oral patient samples [7], and Rosenbach et al. described the simultaneous expression of genes associated with rapid growth and stress response during gut colonization—overlapping signatures rarely observed in vitro [14]. During infection of zebrafish, the expression pattern of *C*. *albicans* reflected distinct phases, with filamentation dominating in the invasive phase and iron acquisition dominating in the damage phase [15]. Using SAGE, Hu et al. observed increased expression of genes encoding transporters and stress-response proteins by *C*. *neoformans* cells infecting lungs of mice, indicating a nutrient-limited and hostile host environment [10]. Similarly, McDonagh et al. used microarrays to define *A*. *fumigatus* genes essential for adaptation to lung tissue [11]. The same laboratory elucidated the role of alkaline adaptation in *Aspergillus* lung infections by an in vivo time series from a pH-adaptation defective mutant (Δ*pacC*) and its wild type and identified the transcription factor Rim101/PacC as a regulator of cell wall biosynthetic genes [17]. Similar observations were made by O’Meara et al. for Rim101/PacC from *C*. *neoformans* during cryptococcal lung infections [21]. Xu et al. observed transcriptional responses to metal limitation in the early stages and response to phagocytosis by macrophages in later stages of systemic candidiasis by NanoString [3]. Using RNASeq, Bruno et al. proposed a role for the inflammasome in the immunopathogenesis of vulvovaginal candidiasis [23]. Chen et al. investigated the transcriptome of *C*. *neoformans* at sites of human meningitis by RNAseq [25]. The RNA pattern reflected surprising metabolic activity within CSF from patients, which was comparable to growth in complex medium. Munoz et al. investigated the gene expression of *Blastomyces* during pulmonary infections of mice by using RNAseq and observed a strong response against reactive oxygen species (ROS) and zinc limitation, among others [24]. As expected, it was demonstrated that the dermatophyte *Arthrodema benhamiae* expressed a great variety of different protease genes on the skin of rodents [16]. However, against expectation, the pattern of protease genes was very different from growth on keratin in vitro. Finally, when comparing the *C*. *albicans* transcriptome during infections of mice and an alternative invertebrate model, Amorim-Vaz et al. found a surprisingly conserved “core” virulence program in these diverse hosts, which included stress response, iron acquisition, and biofilm formation [2]. As can be seen from this list, which is far from exhaustive, many transcriptional patterns appear seemingly independent of model and infecting fungus.

## Conclusion

In the majority of in vivo studies, infection-associated expression profiles have been linked to more-or-less specific, defined processes and have identified recurrent patterns of fungal activities associated with infection. These include (1) niche-specific metabolism [3,5,10,12–14,16,19]; (2) acquisition of nutrients, in particular metals [3,6,11,15,20,24,25]; (3) oxidative stress responses [12,19,25]; (4) pH adaptation [6,11,17,21]; and (4) morphological transitions [6,7,12,15,24]. These findings suggest that the host is a hostile environment from the pathogen's point of view—in line with our expectations.

So, do in vivo transcriptomes simply tell us that what we measure in vivo is similar to what we observe in cultures and reaction tubes? The answer is yes and no. As discussed above, in vitro data can be very similar to in vivo data, but also very different in central aspects.

For example, while the RNA pattern of *C*. *neoformans* during human meningitis was comparable to growth in complex medium [25], more than 1,000 *A*. *fumigatus* genes were found as differentially expressed only in vivo and not in any infection-simulating in vitro condition [11].

The discovery that many of the adaptive responses that we measure on the bench indeed occur during infection is an important one in itself—evidently, our models do reflect actual aspects of infection. However, in vivo transcriptional profiles also show us that some of our predictions and in vitro models are misleading. Equally, or even more significantly, the complexity of infection processes can go well beyond observations made with simple in vitro models. Combinations of stresses, temporal and spatial separation of transcriptional responses in subpopulations, and the (often reciprocal) dynamics of a two-species system influence the overall transcriptome in in vivo samples.

This may explain why we find not only predicted but also surprising new transcriptional patterns, which are not observed under laboratory conditions. These host-specific patterns deserve our future attention, as they may provide us with data on infection-specific processes, which do not occur in cultures and reaction tubes.

Exploiting these emerging in vivo datasets, although challenging, will have great potential for the development of future diagnostic and therapeutic options. Currently, we have not yet found clear molecular biomarkers to detect and discriminate between different fungal infections. So far, we have found only few factors essential for virulence (but not for standard in vitro growth) as potential new drug targets. However, recent years have seen not only promising new technical advances in the extending field of in vivo transcriptional profiling, but also the advent of many sophisticated bioinformatical tools. The latter, especially, will allow us to gather new and meaningful information from increasingly larger and more complex datasets. The era of in vivo transcriptional profiling has only just begun.
